# Comparison of Contemporary and Historic Highly Pathogenic Avian Influenza A(H5N1) Virus Replication in Human Lung Organoids

**DOI:** 10.3201/eid3102.241147

**Published:** 2025-02

**Authors:** Meaghan Flagg, Brandi N. Williamson, Johan A. Ortiz-Morales, Tessa R. Lutterman, Emmie de Wit

**Affiliations:** National Institute of Allergy and Infectious Diseases, National Institutes of Health, Hamilton, Montana, USA

**Keywords:** Influenza A virus, influenza, zoonoses, respiratory infections, viruses, highly pathogenic avian influenza, H5N1, clade 2.3.4.4b, outbreak, human lung organoids

## Abstract

We compared virus replication and host responses in human alveolar epithelium infected with highly pathogenic avian influenza (HPAI) A(H5N1) viruses. A/Vietnam/1203/2004 replicated most efficiently, followed by A/Texas/37/2024, then A/bovine/Ohio/B24OSU-342/2024. Induction of interferon-stimulated genes was lower with A/Texas/37/2024 and A/bovine/Ohio/B24OSU-342/2024, which may indicate a reduced disease severity of those viruses.

Clade 2.3.4.4b highly pathogenic avian influenza (HPAI) A(H5N1) viruses have circulated in avian species in North America since 2021. Subsequently, those viruses have been detected in a wide range of mammal species (*1*). In 2024, clade 2.3.4.4b HPAI H5N1 virus was detected in dairy cattle, in both tissue samples and milk from infected animals (*2*), and then spread to multiple herds in 16 US states (https://www.aphis.usda.gov/livestock-poultry-disease/avian/avian-influenza/hpai-detections/hpai-confirmed-cases-livestock). The broadened host range of clade 2.3.4.4b H5N1 viruses and unprecedented levels of transmission to mammals has raised concerns about potential spillover into humans.

By January 6, 2025, the Centers for Disease Control and Prevention had confirmed 66 human cases of HPAI H5N1 virus infection in the United States (https://www.cdc.gov/bird-flu/situation-summary/index.html). Many of those cases were linked to exposure to infected cattle. However, recent outbreaks in Colorado have resulted in identification of additional human cases linked to infected poultry (*3*). Virus isolated from a worker at a dairy farm in Texas (A/Texas/37/2024) was shown to be closely related to viruses circulating in cattle, suggesting that this case was likely a result of direct cow-to-human transmission (*4*). Reported human symptoms included conjunctivitis, 1 person reported mild respiratory symptoms (*5*) , and 1 person died (https://ldh.la.gov/news/H5N1-death). Those symptoms starkly contrast prior HPAI H5N1 virus infections in humans, which resulted in severe respiratory disease and death in nearly 50% of cases (*6*). To assess the risk for developing severe disease after infection with contemporary HPAI H5N1 virus, we evaluated virus replication, host cell survival, and induction of innate immune responses in human alveolar epithelium infected with A/Texas/37/2024 or cattle isolate A/bovine/Ohio/B24OSU-342/2024, compared with a historic H5N1 isolate (A/Vietnam/1203/2004) derived from a fatal human case in 2004 (*7*).

## The Study

Virus replication and host cell damage in the alveolar epithelium are key drivers of severe respiratory disease. Human lung organoids are a physiologically relevant state-of-the-art model of primary human alveolar epithelium. Lung organoids consisting of alveolar type 2 (AT2) epithelial cells can be cultured from adult stem cells isolated from lung tissue (*8*,*9*) or from induced pluripotent stem cells (iPSCs) differentiated into AT2 cells (*10*). Both model systems accurately recapitulate the fitness and pathogenicity of respiratory viruses as observed in humans (*8*,*9*,*11*,*12*). We infected AT2 cells from both iPSC-derived human lung organoids (ihLOs) and adult stem cell–derived human lung organoids (hLOs) (human donor lung tissue provided by Chuong D. Hoang and Nathanael Pruett, National Cancer Institute [NCI], National Institutes of Health [NIH], Bethesda, MD, USA). Deidentified human lung tissue samples were collected in accordance with institutional review board–approved protocols at the NIH Clinical Center. We infected ihLOs and hLOs with 3 HPAI H5N1 isolates and compared virus replication, host cell survival, and innate immune responses over time.

**Figure 1:**
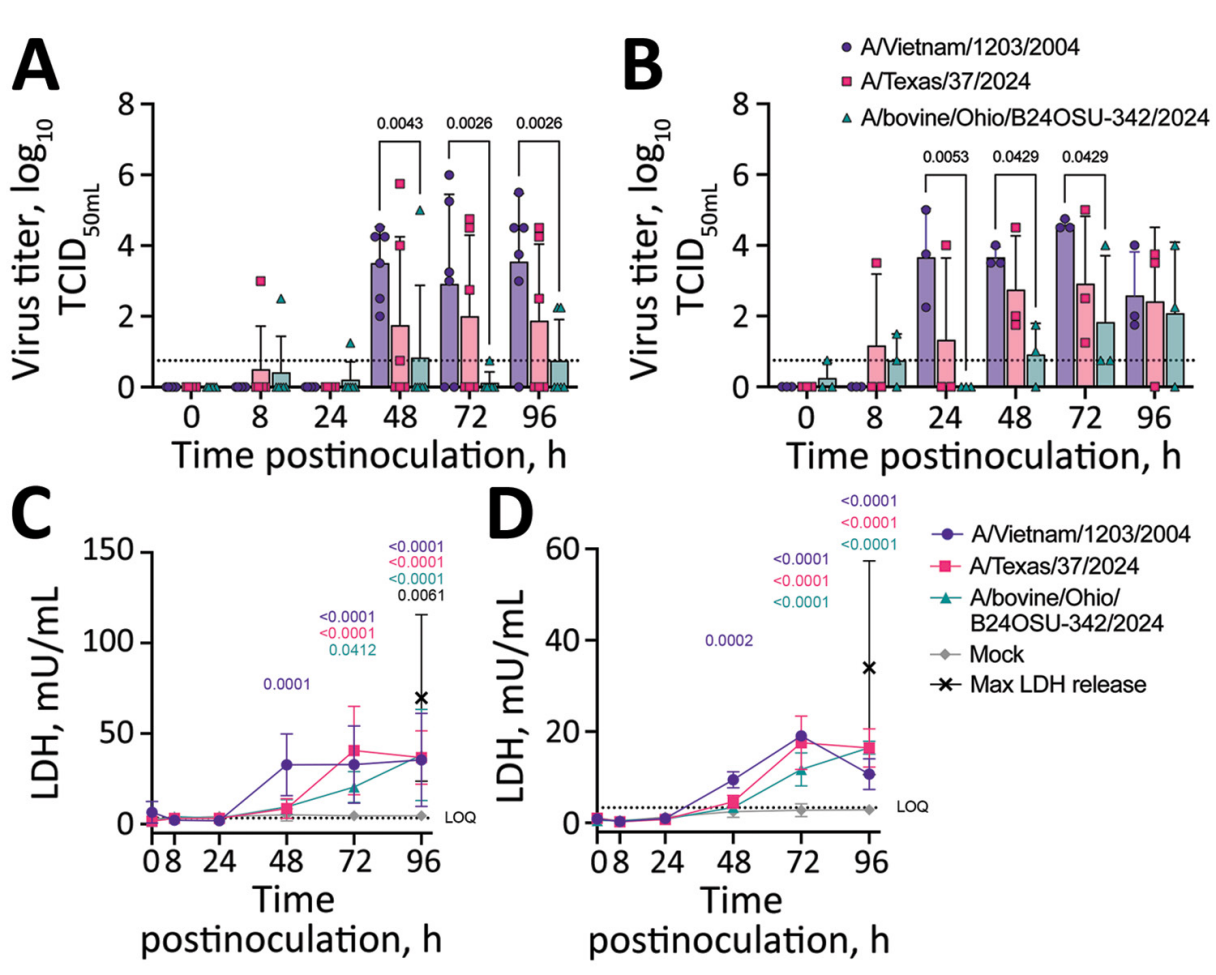
Virus replication and cytotoxicity in a comparison of contemporary and historic highly pathogenic avian influenza A(H5N1) virus replication in human lung organoids. A, B) Virus replication in ihLO (A) hLO (B). Cells were infected at a multiplicity of infection of 0.1 with 1 of the 3 indicated HPAI H5N1 virus isolates. Organoids were removed from Matrigel (Corning, https://www.corning.com) and incubated with virus for 1 hour at 37°C, after which virus was removed, organoids were washed, and replated in Matrigel. Culture supernatant samples were taken at 0, 8, 24, 48, 72, and 96 hours postinoculation and titered on Madin-Darby canine kidney cells. Titrations were read after 3 days by using a hemagglutination assay using turkey red blood cells. Dashed line indicates lower limit of detection; error bars denote means and SDs. p values <0.05 are indicated above bars. Of note, ihLOs and hLOs were derived from different donors. C,D) Cytotoxicity in ihLO (C) hLO (D). Culture supernatants from ihLOs (C) or hLOs (D) were collected and tested for release of lactate dehydrogenase into culture supernatant according to the LDH-Glo Cytotoxicity Assay protocol (Promega, https://www.promega.com) as an indicator of cell death. Results were converted to mU per mL LDH determined by the standard curve using a simple linear regression. Cells lysed with Triton X-100 (MilliporeSigma, https://www.sigmaaldrich.com) were included as maximum LDH release controls. Dashed line indicates lower limit of quantification. Error bars denote means and SDs of ihLO (n = 6 ) or hLO (n = 3) biologic replicates. Statistical analysis was conducted using 2-way analysis of variance followed by Tukey posttest. p values <0.05 are indicated and color coded by isolate. hLOs, adult stem cell–derived human lung organoids; HPAI, highly pathogenic avian influenza; ihLO, iPSC-derived human lung organoids; LDH, lactate dehydrogenase; iPSCs, induced pluripotent stem cells.

We found that the isolate A/Vietnam/1203/2004 replicated to higher titers in both ihLOs and hLOs (Figure 1, panels A, B) compared with cattle isolate A/bovine/Ohio/B24OSU-342/2024 (provided by Richard Webby, St. Jude Children’s Research Hospital, Memphis, TN, USA, and Andrew Bowman, Ohio State University, Columbus, OH, USA). Of note, we detected a trend toward increased replication of A/Texas/37/2024 (provided by Todd Davis, CDC, Atlanta, GA, USA), compared with the bovine isolate, suggesting enhanced fitness of that virus in human cells compared with its predecessors circulating in cattle.

To evaluate potential pathogenicity of the contemporary HPAI H5N1 viruses, we quantified cell death over time in infected lung organoids. We observed cell death earlier in organoids infected with the A/Vietnam/1203/2004 isolate (Figure 1, panels C, D). Infection with the A/Texas/37/2024 and A/bovine/Ohio/B24OSU-342/2024 isolates also resulted in cell death, but at later timepoints (72–96 hours postinoculation). That finding is in accordance with the slower replication kinetics observed with those viruses compared with the A/Vietnam/1203/2004 isolate (Figure 1, panels A, B).

**Figure 2:**
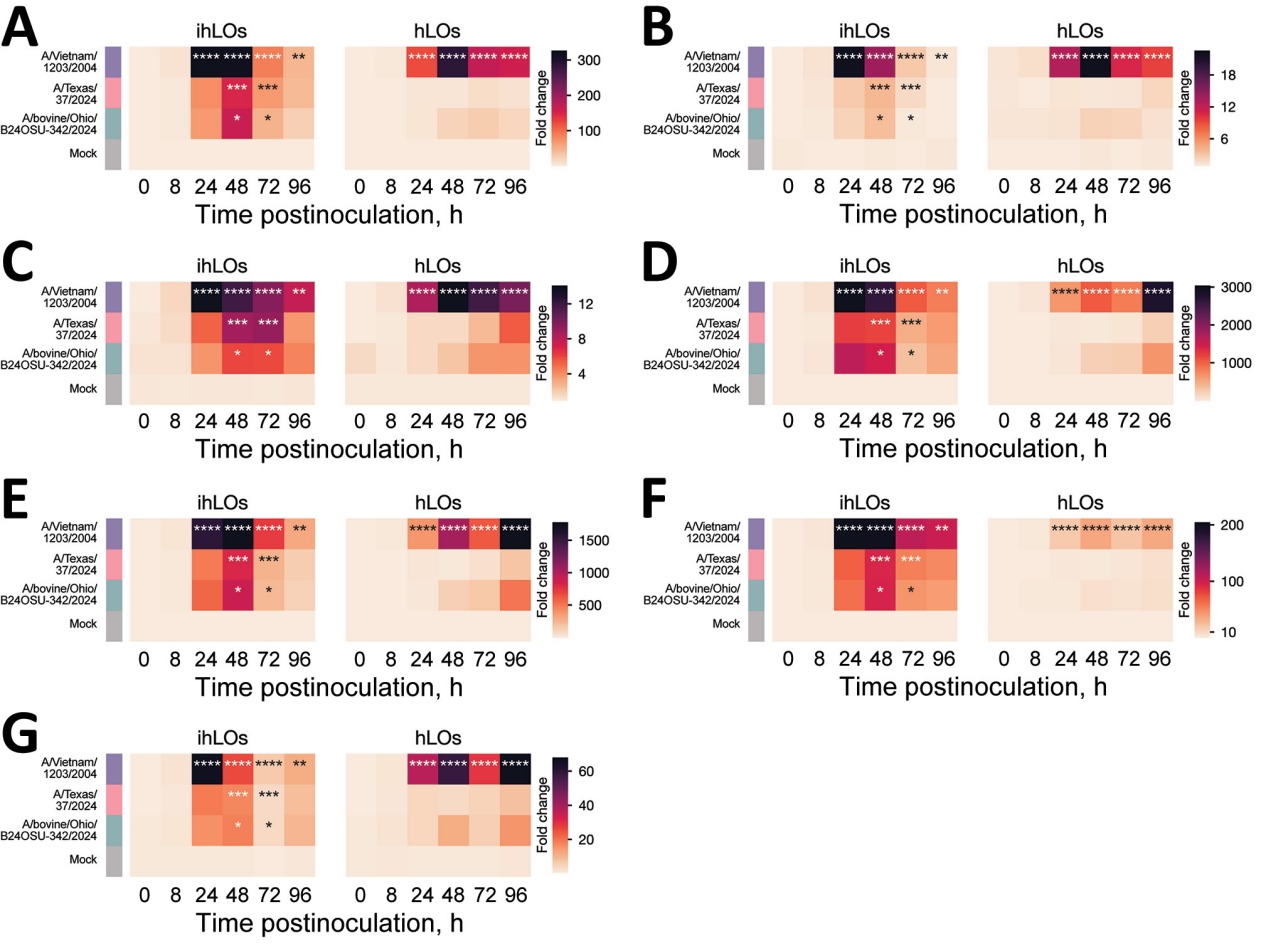
Induction of ISGs in a comparison of contemporary and historic highly pathogenic avian influenza A(H5N1) virus replication in human lung organoids. A) ISG15; B) ISG20; C) interferon-induced transmembrane 3; D) interferon-induced protein with tetratricopeptide 1; E) myxovirus resistance 1; F) 2′-5′-oligoadenylate synthetase 1; G) retinoic acid–inducible 1. We infected ihLO and hLO as described in Figure 1. We extracted RNA from 2.5 × 10^4^ cells by using the QIAGEN RNeasy kit (QIAGEN, https://www.qiagen.com) following the tissue extraction instructions. We ran quantitative reverse transcription PCR by using primers (Integrated DNA Technologies, https://www.idtdna.com) to detect ISGs. Data were normalized to an internal control (ACTB), and fold change was calculated relative to timepoint-matched mock-infected controls. Mean fold change is reported for 6 ihLO and 3 hLO biologic replicates. Statistical analysis was performed using 2-way analysis of variance followed by Dunnett posttest; p values <0.05 for comparisons of infected versus mock samples are indicated. hLOs, adult stem cell–derived human lung organoids; HPAI, highly pathogenic avian influenza; ihLO, iPSC-derived human lung organoids; iPSCs, induced pluripotent stem cells; ISGs, interferon stimulated genes; LDH, lactate dehydrogenase.

**Figure 3:**
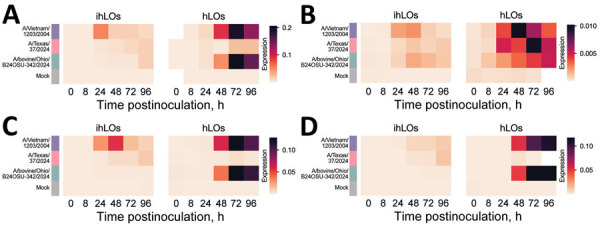
Induction of pro-inflammatory cytokines in a comparison of contemporary and historic highly pathogenic avian influenza A(H5N1) virus replication in human lung organoids. A) interferon β; B) tumor necrosis factor α; C) interleukin (IL) 6; D) IL-1β. iPSC-derived human lung organoids (ihLO) or adult-derived human lung organoids (hLO) were infected as described in Figure 1. We infected ihLO and hLO as described in Figure 1. We extracted RNA from 2.5 × 10^4^ cells by using the QIAGEN RNeasy kit (QIAGEN, https://www.qiagen.com), following the tissue extraction instructions. We ran quantitative reverse transcription PCR by using primers (Integrated DNA Technologies, https://www.idtdna.com) to detect proinflammatory cytokines. Data were normalized to an internal control (ACTB), and expression was calculated using the 2^–ΔCt^ method, due to limited detection of proinflammatory cytokines in mock-infected samples. White cells indicate samples where PCR amplification was not detected. Mean expression for 6 ihLO and 3 hLO biologic replicates is shown. hLOs, adult stem cell–derived human lung organoids; ihLO, iPSC-derived human lung organoids; iPSCs, induced pluripotent stem cells.

We quantified induction of interferon-stimulated genes (ISGs) ISG15, ISG20, interferon induced transmembrane 3, interferon-induced protein with tetratricopeptide 1, myxovirus resistance 1, 2′-5′-oligoadenylate synthetase 1, and retinoic acid-inducible 1 (Figure 2) and proinflammatory cytokines interferon β, tumor necrosis factor α, interleukin 6, and interleukin 1B (Figure 3) by quantitative reverse transcription PCR as a measure of the host innate immune response to infection. ISG induction was highest in organoids infected with the A/Vietnam/1203/2004 isolate. That result was most pronounced in the adult stem cell–derived hLOs, in which ISG induction was not detected in organoids infected with the A/Texas/37/2024 or A/bovine/Ohio/B24OSU-342/2024 isolates, despite the presence of replicating virus. We observed moderate ISG induction in ihLOs infected with the A/Texas/37/2024 and A/bovine/Ohio/B24OSU-342/2024 isolates. We observed a different pattern of induction for proinflammatory cytokines; detection was largely confined to A/Vietnam/1203/2004-infected ihLOs, and A/Vietnam/1203/2004-infected and A/bovine/Ohio/B24OSU-342/2024–infected hLOs (Figure 3).

The unusual transmission of clade 2.3.4.4b HPAI H5N1 viruses to mammals has raised concerns about the risk for spillover into the human population, and the possibility of outbreaks leading to severe disease. We assessed virus replication and host responses in human alveolar epithelium because virus replication and host cell damage in that site is a key driver of severe respiratory disease. The reduced replication levels of the contemporary A/Texas/37/2024 and A/bovine/Ohio/B24OSU-342/2024 isolates in lung organoids compared with the historic A/Vietnam/1203/2004 isolate could explain why recent human influenza cases involving the clade 2.3.4.4b viruses resulted in mild illness (*4*,*6*), as opposed to the severe respiratory disease associated with previous HPAI outbreaks in Vietnam (*13*,*14*). The presence of a lysine at position 627 in the polymerase basic (PB) 2 protein has been associated with adaption of avian influenza viruses to mammal hosts and is known to increase virus replication in the mammalian respiratory tract (*15*). That substitution (E627K) is in both the A/Vietnam/1203/2004 and A/Texas/37/2024 viruses but not the A/bovine/Ohio/B24OSU-342/2024 isolate (*4*), which could explain the increased replication observed for the A/Texas/37/2024 isolate compared with the bovine isolate.

Another factor contributing to the reduced disease severity in humans after infection with clade 2.3.4.4b viruses compared with previous HPAI H5N1 virus cases may be differential activation of the immune system. We observed substantially higher induction of ISGs in lung organoids infected with the A/Vietnam/1203/2004 isolate. An overly exuberant immune response, including cytokine storm, is known to play a role in the high mortality rates from HPAI H5N1 virus infections observed during the 2003 and 2004 outbreaks in Vietnam (*14*). Reduced ISG induction elicited by the A/Texas/37/2024 and A/bovine/Ohio/B24OSU-342/2024 isolates despite detection of virus replication might indicate that those viruses have further adapted to counteract the interferon system in humans, possibly due to their more extensive circulation in mammals. That limited innate immune activation may contribute to their reduced pathogenicity along with other factors, such as differences in prior immunity.

Despite differences in virus replication and ISG induction, we observed similar levels of cell death by 96 hours postinoculation for all 3 viruses. Previous work has shown that direct virus-induced cytotoxicity is not always indicative of pathogenicity in vivo because cytotoxicity was not observed in SARS-CoV-2–infected lung organoids (*11*), despite the ability of that virus to cause severe respiratory disease. Taken together, those data suggest that epithelial-extrinsic factors, possibly related to immune activation, govern pathogenicity in vivo.

## Conclusions

In summary, this study provides a characterization of virus replication and host responses to infection in human alveolar epithelium between a contemporary clade 2.3.4.4b human HPAI H5N1 isolate and the highly virulent A/Vietnam/1203/2004 virus. Further studies are warranted to understand how these viruses interact with the innate immune system, particularly regarding differential ISG and proinflammatory cytokine induction, and how this affects pathogenesis in vivo. Nonetheless, our results indicate that the clade 2.3.4.4b HPAI viruses currently circulating in cattle will likely exhibit reduced human disease severity compared with historic HPAI viruses but should be closely monitored for changes that may influence pathogenicity or transmissibility.
