# Correction: Vol. 31, No. 2

**DOI:** 10.3201/eid3105.C33105

**Published:** 2025-05

**Keywords:** errata, erratum, correction

Some of the data were inaccurate in Figure 1, panels C and D, in Comparison of Contemporary and Historic Highly Pathogenic Avian Influenza A(H5N1) Virus Replication in Human Lung Organoids (M. Flagg et al.). The article has been corrected online (https://wwwnc.cdc.gov/eid/article/31/2/24-1147_article).

